# When Stroke Disguises as Dementia: A Case of Missed Cerebral Venous Thrombosis

**DOI:** 10.1002/ccr3.70038

**Published:** 2025-01-10

**Authors:** Tina Moghadam Fard, Mehrnaz Hosseinzadeh, MohammadAli Shokri, Mostafa Almasi‐Dooghaee, Fatemeh Sadat Mirfazeli

**Affiliations:** ^1^ Cellular and Molecular Research Center, Faculty of Advanced Technologies in Medicine Iran University of Medical Sciences Tehran Iran; ^2^ National Brain Centre Iran University of Medical Sciences Tehran Iran; ^3^ Mental Health Research Center, Psychosocial Health Research Institute Iran University of Medical Sciences Tehran Iran; ^4^ Department of Neuroscience, Faculty of Advanced Technologies in Medicine Iran University of Medical Sciences Tehran Iran; ^5^ Neurology, Firoozgar Hospital, School of Medicine Iran University of Medical Tehran Iran; ^6^ Faculty of Advanced Technologies in Medicine Iran University of Medical Sciences Tehran Iran; ^7^ Mental Health Research Center, Psychosocial Health Research Institute, Department of Psychiatry, School of Medicine Iran University of Medical Sciences Tehran Iran

**Keywords:** cerebral venous thrombosis, cerebrovascular accident, dementia, vascular dementia, Wernicke aphasia

## Abstract

Cerebrovascular thrombosis is among the most critical medical conditions, making early diagnosis and management crucial. Although some symptoms of cerebrovascular thrombosis are typical and lead to early diagnosis, they can sometimes present with rare and unusual symptoms, complicating the diagnostic process. Given the morbidity and mortality associated with these events, it is important to be aware of unexpected symptoms to diagnose and manage these patients more accurately and rapidly. We report a 74‐year‐old female initially misdiagnosed with Alzheimer's because of cognitive decline and disorganized speech. Her symptoms did not improve with Alzheimer's treatment. She was reevaluated by a neurologist, and her cognitive test results were impaired. Her brain MRI revealed a previously undetected left transverse sinus cerebral venous thrombosis with subcortical white matter lesions. The patient was managed acutely with subcutaneous enoxaparin and transitioned to oral rivaroxaban, resulting in significant improvement. This case report aimed to draw attention to the pitfalls of diagnosing dementia‐like syndromes in the elderly, advocating for a systematic approach to differential diagnosis. It emphasizes that a collaborative effort between psychiatrists, neurologists, radiologists, and other healthcare members is essential for accurate diagnosis and timely intervention, which can significantly alter the management and outcome for the patient.

**Summary**

- Cognitive decline can occur in cerebrovascular accidents but is rare in cerebral venous thrombosis, leading to potential misdiagnosis.
- Recognizing CVT as a reversible cause of dementia‐like symptoms is crucial. Accurate diagnosis through a multidisciplinary approach is critical, as timely treatment of CVT can lead to significant recovery.

## 1 Introduction

The accurate diagnosis of cognitive decline in the elderly is a complex puzzle that demands careful consideration of various potential etiologies. According to previous studies, Alzheimer's disease, as the most common type of dementia, affects 3.2% of the population in Iran. The clinical presentation of progressive cognitive decline often leads clinicians toward a neurodegenerative diagnosis, like Alzheimer's disease [1, 2]. However, dementia, more specifically those presented with rapidly evolving progression or with reversible symptoms, have many other causes that clinicians should keep in mind.

Cerebrovascular accidents (CVAs), with a respective national prevalence and incidence rate of 1.11% and 0.12% in Iran, can be due to either hemorrhagic or ischemic events, with both types presenting distinct challenges in management and prognosis [3]. Cerebral venous thrombosis (CVT) with a reported annual frequency of 0.0012% is a type of stroke that may have both hemorrhagic or ischemic characteristics in imaging and have some unique clinical features like headache, seizure, papilledema, encephalopathy, or cranial neuropathy [4, 5, 6]. Aphasia, a communication disorder arising from damage to the regions of the brain responsible for language, that is, Wernicke's (Brodmann area 22) and Broca's (Brodmann area 44), is a common consequence of stroke, presenting in 20%–40% of stroke survivors during the initial period following the event [7, 8, 9, 10]. Its impact on quality of life is multifaceted and substantial [7, 8, 11].

Wernicke's aphasia, also called receptive aphasia or fluent aphasia, is a language disorder characterized by impaired ability to understand spoken words and sentences, coupled with fluent but nonsensical speech and a lack of awareness of these communication deficits [12]. It typically results from damage to the posterior portion of the temporal lobe in the language‐dominant hemisphere of the brain [9]. Wernicke's aphasia often not only results from ischemic stroke but can also arise from head trauma, infections, seizures, and other neurological disorders [10, 13, 14].

This case report details the diagnostic journey of a 74‐year‐old Iranian female whose initial symptoms were suggestive of dementia, including disorganized speech characteristic of Wernicke's aphasia, accompanied by behavioral disturbances and a notable decline in activities of daily living.

## 2 Case History/Examination

**FIGURE 1:**
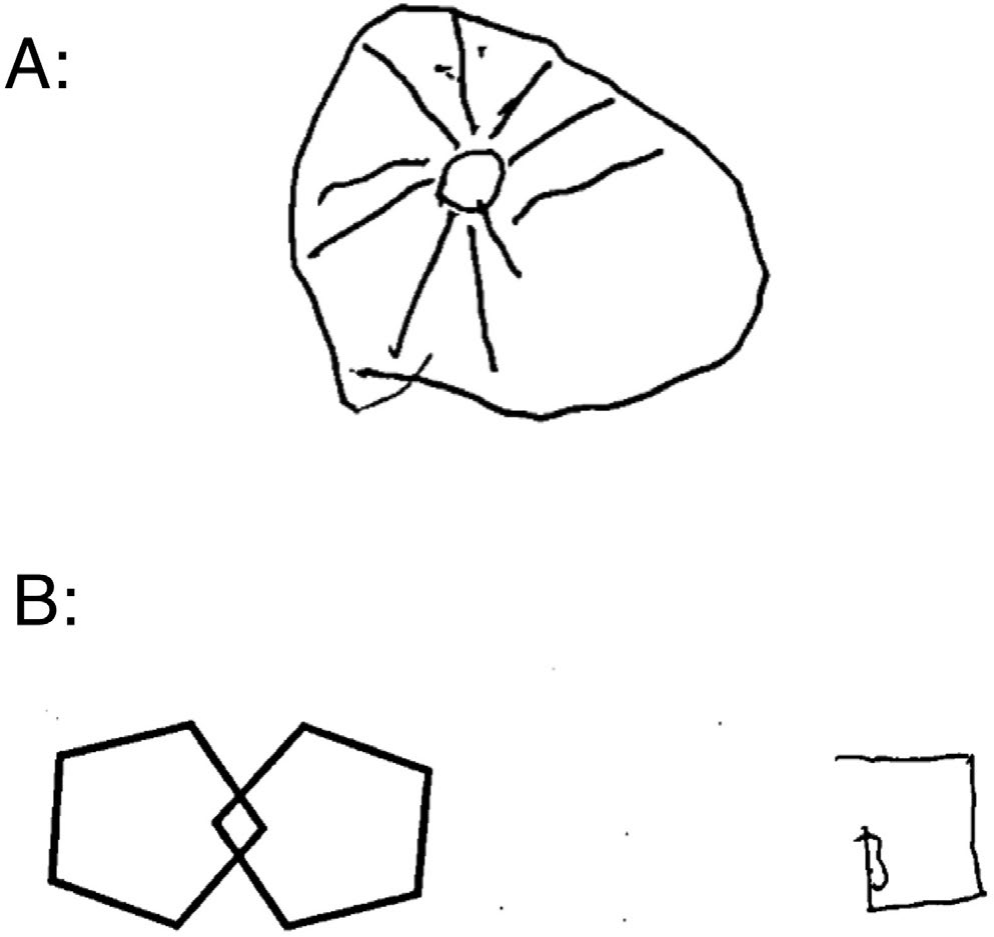
Clock Drawing and Crossed‐Pentagon Drawing tests. (A) Clock Drawing Test revealed a perseveration error. The patient did not stop drawing the clock hands, and continued to draw straight lines until stopped by the examiner. (B) Crossed‐Pentagon Drawing Test revealed marked impairment in visuospatial construction.

A 74‐year‐old right‐handed female, a widowed homemaker, presented to our psychiatry clinic on the August 1, 2023. Her chief complaints were a sudden onset of disorganized speech, severe anxiety, and self‐harm behaviors that had begun abruptly on March 27, 2023, when she and her family had gone on a trip out of town. The first example of her disorganized speech was a nonsensical phrase in Persian: “همسایه ما رفته کفاشی قورمه سبزی بخره,” which translates to English as “Our neighbor has gone to the cobbler to buy Ghormeh Sabzi.” The patient's speech was characterized by rapid and incoherent word production, suggestive of Wernicke's aphasia. These symptoms were accompanied by agitation, anxiety, and aggression, which fluctuated in severity but progressively worsened over the following months. Notably, her condition deteriorated to the extent of experiencing urinary and fecal incontinence and an incessant urge to elope from her residence. The patient had a psychiatric history of depression, managed with sertraline 100 mg daily for the past 10 years. Her medical and surgical histories were otherwise unremarkable, with no known history of cognitive decline or neurological disorders. The patient had a history of myocardial infarction in her sister but no family history of genetic, psychiatric, or neurological diseases. Upon psychiatric evaluation, the patient demonstrated time disorientation, anomia, persecutory delusions, and both visual and auditory hallucinations. In the Clock Drawing Test, a simple, safe, and time‐efficient assessment widely used in clinical settings for evaluating cognitive function, patients are asked to draw the face of a clock, all the numbers and hands set to a specific time. This task requires a range of cognitive abilities [15]. The patient demonstrated perseveration, characterized by the repetition of an action without an appropriate stimulus, by drawing multiple clock hands. Luria's three‐step test, also known as the fist‐edge‐palm test, is a fast and efficient task for cognitive screening. It requires the patient to perform three specific hand movements [16]. In this case, performance was impaired. Also, the crossed‐pentagons drawing test, which involves drawing two intersecting pentagons to form a rhombus in the overlap [17], was impaired (Figure 1).

The patient underwent a standard battery of cognitive tests, revealing profound deficits:

The Mini‐Mental State Exam (MMSE), introduced by Folstein et al. [18], is a widely used 30‐point tool for assessing cognitive function. It evaluates orientation, registration, attention, calculation, recall, language, and the ability to follow simple commands. In clinical settings, a score of 25 or higher is typically considered normal, helping to detect cognitive impairments [18]. In this case, the score was 2, indicating significant cognitive impairment.

The Addenbrooke's Cognitive Examination—Revised (ACE‐R) is a sensitive and specific 100‐point test used to evaluate cognitive impairment [19]. In this case, the score was 2, possibly due to comprehension difficulties from Wernicke's aphasia.

An initial brain MRI scan, interpreted by a radiologist a few months before (April 24, 2023), was reported as normal—a finding that stood in stark contrast to the severity of the patient's clinical presentation. Consequently, she was diagnosed with Alzheimer's disease and commenced on memantine 14 mg daily and quetiapine 12.5 mg daily. However, her symptoms did not improve as anticipated, prompting a visit to the psychiatric clinic. Because of the acute onset of symptoms and impaired frontal and temporal lobe functions, the patient was referred to our team's neurologist for further assessment.

On the basis of the acute onset of the clinical features and the beginning of the symptoms with Wernicke's aphasia, the possible diagnosis was vascular dementia with involvement of the frontal lobes and the left temporal lobe. This discrepancy between the normal MRI findings and the patient's severe symptoms prompted a thorough reevaluation of the MRI. The medical team, concerned about the unexplained clinical presentation, decided to reassess the initial imaging to ensure no critical details were overlooked. Our neurologist's reevaluation of the patient's brain MRI DICOM images revealed a previously undetected left transverse sinus cerebral venous thrombosis, with some subcortical white matter lesions, but no acute stroke shown in diffusion‐weighted imaging.

**FIGURE 2:**
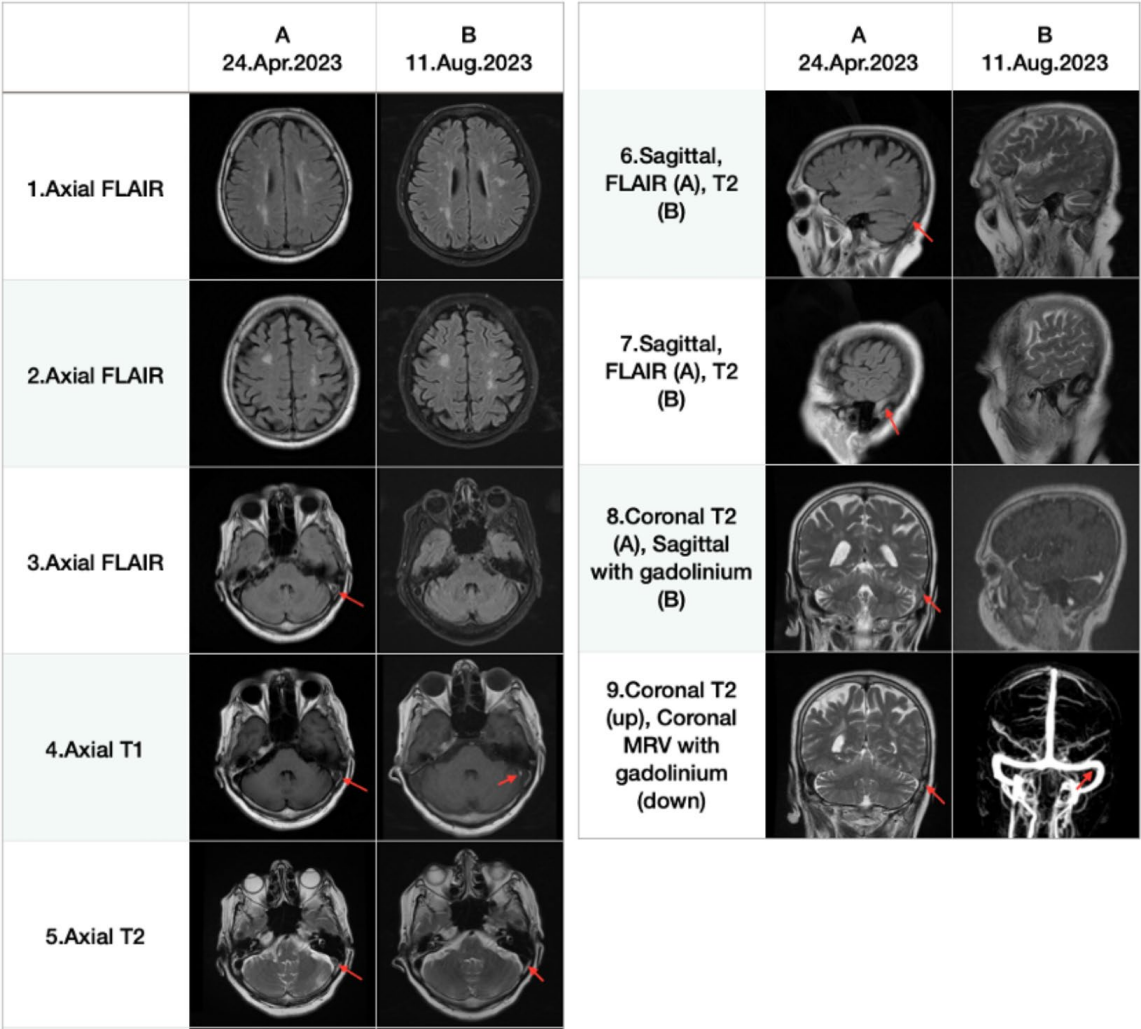
Comparison between the two MR images of the patient taken 7 months apart. In the axial flair images, subcortical white matter lesions were identified in images A1, A2, B1, and B2. A filling defect was observed in the left transverse sinus and sigmoid sinus, spanning images A3–A7, as indicated by a red arrow. Notably, images B3–B7 depict an almost complete resolution of the previously noted defects in the left transverse sinus and sigmoid sinus, with a residual small filling defect persisting in image B4. Additionally, a small filling defect was present in images B8 and B9, with the latter distinctly marked by a red arrow.

Following this new discovery, the patient was hospitalized, and a repeat brain MRI demonstrated several subcortical ischemic lesions, with the most significant located in the right frontal lobe. In addition, the left transverse sinus thrombosis was significantly recanalized, except for a subtle hypersignal area in the left transverse sinus in T1‐weighted imaging. Brain magnetic resonance venography (MRV) demonstrated a diminutive obstruction within the left transverse sinus (Figure 2).

## 3 Differential Diagnosis

On the basis of the patient's age, cognitive decline, and disorganized speech, one initial consideration was dementia, such as Alzheimer's disease. However, the rapid progression, sudden‐onset symptoms, and lack of response to treatment were inconsistent with typical dementia. Additional differential diagnoses included frontotemporal dementia, frontal lobe tumor, and frontal lobe cerebrovascular accident. However, the sudden onset of symptoms reduced the likelihood of frontotemporal dementia, which typically has a more gradual progression. For frontal lobe tumor and frontal lobe cerebrovascular accident, imaging studies can effectively rule out these conditions. The acute presentation suggested the need to explore other causes, including vascular dementia and CVT. Reevaluation of imaging revealed left transverse sinus thrombosis, aligning with her symptoms. This highlights the necessity of considering CVT in atypical cognitive decline cases and underscores the importance of a multidisciplinary approach for accurate diagnosis.

## 4 Conclusion and Results

A multidisciplinary team, including neurologists, psychiatrists, and radiologists, convened to review the case, leading to a consensus that the final diagnosis was a CVT, which manifested with rapidly progressive dementia. The patient was managed acutely with subcutaneous enoxaparin (60 mg every 12 h) and subsequently switched to oral rivaroxaban, which led to an improvement in her general condition.

Her symptoms were reversible, and during her hospital stay, her speech became more organized, and her visual and auditory hallucinations were significantly reduced.

Upon discharge, she was provided with a comprehensive plan for outpatient care and follow‐up. Remarkably, at a follow‐up visit two months post‐discharge, the patient showed a substantial improvement. Her MMSE score had increased to 14 out of 30, and her ACE‐R score to 47 out of 100. Her speech was more organized, and her cognitive and psychiatric symptoms had lessened considerably. The subsequent follow‐up visits revealed progressive improvement in cognitive function and social behavior. After about six months of hospitalization in our hospital, she had approximately independent daily living.

## 5 Discussion

We reported an individual who presented with reversible but rapidly progressive cognitive decline lasting for almost 5 months. The prominent symptom was Wernicke's aphasia, along with discernible behavioral alterations. The patient was diagnosed and approached as a case of rapidly progressive dementia until the family requested further psychiatric evaluation because of worsening behavioral features, visual hallucinations, and persecutory delusions. Initial cognitive evaluations revealed pronounced severity, with a Mini‐Mental State Examination (MMSE) score of 2 out of 30. The diagnostic inference leaned toward vascular dementia owing to the sudden onset and rapidly evolving nature of the disease. Reevaluation of previous imaging studies suggested left transverse sinus thrombosis without gross parenchymal or structural brain lesions. Therapeutic intervention with anticoagulation therapy was promptly initiated, leading to marked improvement in the patient's cognitive status, as evidenced by the subsequent elevation of the Addenbrooke's Cognitive Examination score from 2 to 47 and then to 56. This significant improvement prompts a deeper exploration of the intricate interplay between venous sinus pathophysiology and cognitive derangements and raises questions about the time course and acuity of CVT and anticoagulation therapy.

Cerebrovascular thrombosis, characterized by blood clot formation in cerebral veins or sinuses, typically presents with well‐established diagnostic symptoms. Common manifestations include severe headache in the majority of cases, intracranial hypertension indicated by papilledema, focal neurological deficits, and seizures. These symptoms reflect the impact of compromised blood flow and venous congestion on brain function [20, 21].

From a demographic perspective, cerebrovascular thrombosis tends to be more prevalent among middle‐aged females, with risk factors including pregnancy, estrogen use, hypercoagulation, and brain trauma [21]. These hallmark features and population characteristics have traditionally guided clinicians in diagnosing cerebrovascular thrombosis. Our patient did not have trauma or hormonal exposure but had traveled to a rural area, which may have caused a hypercoagulable state because of dehydration. Cognitive impairments can occur in many survivors of CVT; however, they are not common presenting symptoms [21]. The case under discussion takes a distinctive turn from the norm by presenting a less frequently encountered feature: sudden‐onset cognitive decline.

A thorough exploration of the literature on cerebrovascular thrombosis with cognitive decline as the primary manifestation revealed the rarity of cases resembling the presentation highlighted in this study. Ayele et al. reported a 30‐year‐old female patient with a postpartum presentation of anomia associated with a refractory headache and summarized two other reports with similar presentations explained by CVT [22]. Another reported case was a 40‐year‐old female diagnosed with primary myelofibrosis, presenting with rapidly progressive cognitive decline. However, the patient's symptoms also included focal neurologic deficit of left limb weakness with overt structural brain lesions following multiple dural thromboses and intracranial hemorrhage [23]. A recent study reports a middle‐aged female who presented with neuropsychiatric features comparable to our case, progressing within a few days, alongside notable catatonic features following CVT [24]. A case report discussing CVT as an acute post‐COVID‐19 complication presents a 32‐year‐old female with amnesia followed by headache, with imaging results suggestive of left transverse sinus and superior sagittal sinus thrombosis [25]. Li et al. reported a 62‐year‐old male patient with reversible progressive cognitive decline over 2 months, diagnosed with thrombosis of the left transverse sinus, sigmoid sinus, and jugular venous bulb. The patient's neuroimaging also indicated multiple deep brain lesions, manifested as hypointensity on T1‐weighted MR images [26].

The presented case highlights a diagnostic challenge. Given the patient's advanced age, there was an initial inclination to consider dementia as the main diagnosis, adding complexity to the diagnostic process. Despite deviating from the typical demographic, the distinctive clinical features of our case are even more striking. The patient exhibited severely disrupted cognitive function as the presenting feature, with a unique presentation of functional dementia. This cognitive impairment was associated with very subtle imaging changes that went unrecognized. Contrary to the common perception that dementia necessitates a structural lesion, our case highlights the potential for vascular dementia without an apparent lesion in routine neuroimaging studies. Additionally, the absence of a left temporal lesion raises questions about the observed aphasia. We suppose that the thrombosis in the left transverse sinus may have functionally impaired the function of left temporal lobe, even without any evident anatomic parenchymal abnormality. In summary, this case not only challenges age‐related expectations but also underscores the complexity of cerebrovascular thrombosis presentations, necessitating a reevaluation of diagnostic sensitivity and paradigms.

This report highlights acute, rapidly progressive cognitive impairment as a noteworthy manifestation of CVT. These insights significantly refine our understanding of the condition, prompting a broader and more nuanced diagnostic approach when evaluating patients with suspected cerebrovascular thrombosis, particularly those with unusual chronic and subchronic trajectories. Furthermore, the significance of a multidisciplinary approach cannot be overstated. Collaboration between neurologists, psychiatrists, radiologists, and other specialists is paramount in navigating the complexities of cerebrovascular thrombosis presentations, facilitating accurate diagnosis and optimal patient care. This case not only contributes to the expanding knowledge base of cerebrovascular thrombosis but also underscores the critical role of interdisciplinary collaboration in addressing diagnostic intricacies.

## 6 Conclusion

This case report highlights the challenges of diagnosing dementia‐like syndromes, emphasizing the need for a systematic approach to differential diagnosis. Cognitive decline is a rare presentation of cerebrovascular thrombosis, a critical medical condition requiring early diagnosis and management. It is important to be aware of unexpected symptoms and to foster collaboration among psychiatrists, neurologists, radiologists, and other healthcare professionals to diagnose and manage these patients more accurately and rapidly.

## Author Contributions

**Tina Moghadam Fard:** visualization, writing – original draft, writing – review and editing. **Mehrnaz Hosseinzadeh:** writing – original draft, writing – review and editing. **MohammadAli Shokri:** writing – original draft, writing – review and editing. **Fatemeh Sadat Mirfazeli:** data curation, supervision. **Mostafa Almasi‐Dooghaee:** data curation, supervision.

## Ethics Statement

Ethics approval was obtained from the Iran University of Medical Sciences. Written informed consent was obtained from the patient.

## Consent

Written informed consent was obtained from the patient for the publication of this case report and any accompanying images. A copy of the written consent is available for review by the editor of this journal.

## Conflicts of Interest

The authors declare no conflicts of interest.

## Data Availability

The data supporting the findings of this study are available from the corresponding author upon reasonable request. The data were collected from a single patient.
